# Robust quasi-uniform surface meshing of neuronal morphology using line skeleton-based progressive convolution approximation

**DOI:** 10.3389/fninf.2022.953930

**Published:** 2022-10-27

**Authors:** Xiaoqiang Zhu, Xiaomei Liu, Sihu Liu, Yalan Shen, Lihua You, Yimin Wang

**Affiliations:** ^1^School of Communication and Information Engineering, Shanghai University, Shanghai, China; ^2^Guangdong Institute of Intelligence Science and Technology, Hengqin, China; ^3^School of Computer Engineering and Science, Shanghai University, Shanghai, China; ^4^National Center for Computer Animation, Bournemouth University, Bournemouth, United Kingdom

**Keywords:** neuronal morphology, quasi-uniform mesh, dynamic sculpting, multiresolution techniques, geometry-based techniques, local mapping query, finite-support convolution kernel

## Abstract

Creating high-quality polygonal meshes which represent the membrane surface of neurons for both visualization and numerical simulation purposes is an important yet nontrivial task, due to their irregular and complicated structures. In this paper, we develop a novel approach of constructing a watertight 3D mesh from the abstract point-and-diameter representation of the given neuronal morphology. The membrane shape of the neuron is reconstructed by progressively deforming an initial sphere with the guidance of the neuronal skeleton, which can be regarded as a digital sculpting process. To efficiently deform the surface, a local mapping is adopted to simulate the animation skinning. As a result, only the vertices within the region of influence (ROI) of the current skeletal position need to be updated. The ROI is determined based on the finite-support convolution kernel, which is convolved along the line skeleton of the neuron to generate a potential field that further smooths the overall surface at both unidirectional and bifurcating regions. Meanwhile, the mesh quality during the entire evolution is always guaranteed by a set of quasi-uniform rules, which split excessively long edges, collapse undersized ones, and adjust vertices within the tangent plane to produce regular triangles. Additionally, the local vertices density on the result mesh is decided by the radius and curvature of neurites to achieve adaptiveness.

## 1. Introduction

With a rapid technical development, increasing attention has been paid to biologically detailed brain visualization and simulation. As the most significant information-processing cells in the brain, neurons are made up of dendrites and axons, which are responsible for receiving and sending signals, respectively. Each neuron is an electrical entity and various methods have been proposed to simulate the electrical behavior of neurons. However, due to the complexity of neuronal structure, most of these methods simulate the conduction behavior of electrical and biochemical signals in a low-dimensional space. Recently, there is a growing requirement to simulate cellular behavior based on 3D high-fidelity models (Mörschel et al., 2017). Since numerical simulation of a reaction-diffusion problem requires a fully-defined neuronal geometry, a key challenge in neuroscience is to robustly generate a high-quality neuronal membrane surface, which can be used to create tetrahedrons for reaction-diffusion simulation in full neurons or networks of neurons.

From a morphological point of view, the anatomy of neurons can be obtained by interactively tracing neuron elements from microscope images, or automatically extracted using a series of software tools (Wang et al., 2019; Chen et al., 2022). The neuronal morphology tracking program usually provides a tree-like structure: the unique morphological point in the center of a soma serves as the root node, and the morphology of the neurite is composed of an ordered sequence of interconnected nodes. Besides the 3D coordinates of the skeletal nodes, thickness at each node can also be included to form a point-and-diameter structure. The anatomical features of neurons captured through morphological reconstruction can be used for detailed electrophysiological simulations of voltage dynamics throughout the 3D structure of the neuron (Wilson et al., 1988; Carnevale and Hines, 2006; Gleeson et al., 2007). Unfortunately, the point-and-diameter approximation becomes a problem when one wishes to use the morphologies for visualization or simulation. Thus, the visualization of such spatiotemporal data poses a particular challenge because of the complexity of neuron morphologies and the limitations of the morphological point-and-diameter representations.

**Figure 1:**
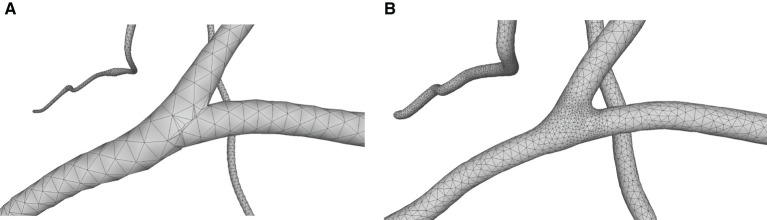
**(A)** Non-adaptive mesh generation with ill-shaped triangles by traditional approaches. **(B)** High quality mesh generation with adaptive vertex densities by the proposed approach.

For the neuronal morphologies of tree-like structures, branch modeling is usually the most complicated part of producing neuronal membrane surfaces, as a self-intersection tends to occur at branches. However, we note that smooth branch ramifications are not taken into consideration in most of previous approaches, and the majority of them concentrate on uniform remeshing, which try to generate a whole mesh with a same edge length (Vorsatz et al., 2003; Botsch and Kobbelt, 2004; Kil et al., 2006; Stanculescu et al., 2011). To be able to represent fine geometric details, these approaches inevitably place too many triangles in regions with low-curvatures. To solve these problems, a novel approach is proposed in this paper for robust neural morphology modeling based on skeleton-driven progressive adaptive remeshing, which achieves both smooth surface approximation and high-quality mesh vertex distributions at the branches (see Figure 1).

**Figure 2:**
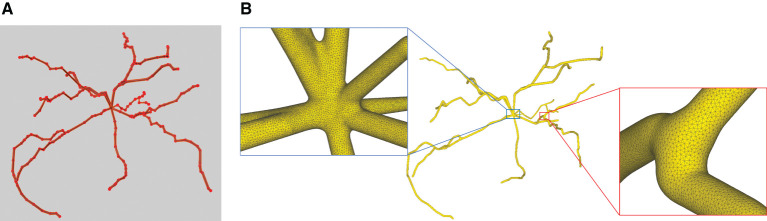
A basal ganglia neuron acquired from NeuroMorpho.Org. **(A)** The point-and-diamter representation. **(B)** The generated mesh representation.

**Figure 3:**
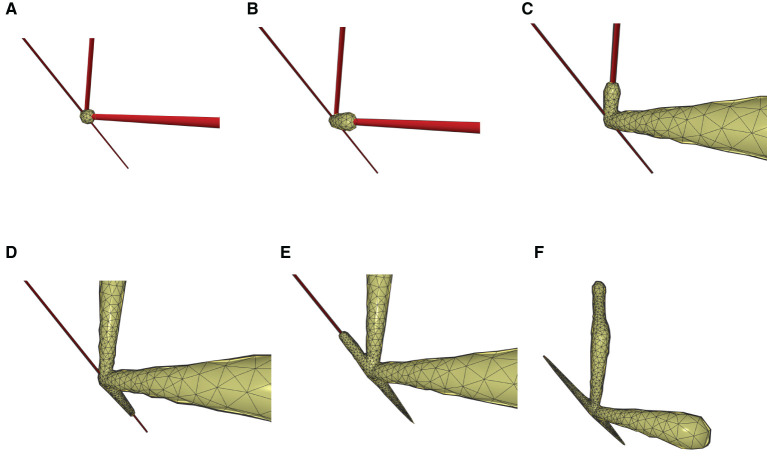
The iterative deformation process. **(A)** An initial spherical mesh placed at the root node. **(B–F)** Generation of the gradually evolved mesh structure driven by the neuronal skeletons.

This paper presents a sculpting-like mesh generation method for 3D visualization of neuron surface and reaction-diffusion simulation applications. A pair of neuronal skeleton and surface mesh are illustrated in Figure 2. In the presented method, the skeletons are firstly loaded from neuronal morphology files, which are then used to drive iterative deformations of the initial triangular mesh placed at the root node (see Figure 3). This method allows subsequent adaptive mesh refinements according to different geometric details and creates high-quality neuronal membranes robustly. Additionally, the watertight nature of our surface can also be used to create tetrahedral volumes for stochastic reaction-diffusion simulations. In summary, the main contributions of this paper are as follows:

1. A high-quality neuronal membrane modeling approach is proposed, and the topological robustness can be easily guaranteed through a progressive surface evolution, as the shape of each updated triangle satisfies the adopted quasi-uniform remeshing rules which result in regular triangles.
2. A local convolution surface approximation is presented to model branching neuron morphology and achieve a pleasing smoothness and efficiency of the progressive mesh evolution.
3. Procedural adaptive mesh refinements are utilized to capture varying scales of geometric details, which balances the mesh quality and the computational expense.
4. A skeletal vertex mapping scheme is introduced to accelerate the neighboring queries, and also to relate mesh vertices to the original morphological skeleton, which is a requirement for other visualization and simulation applications.

## 2. Methods

### 2.1. Overall workflow

**Figure 4:**
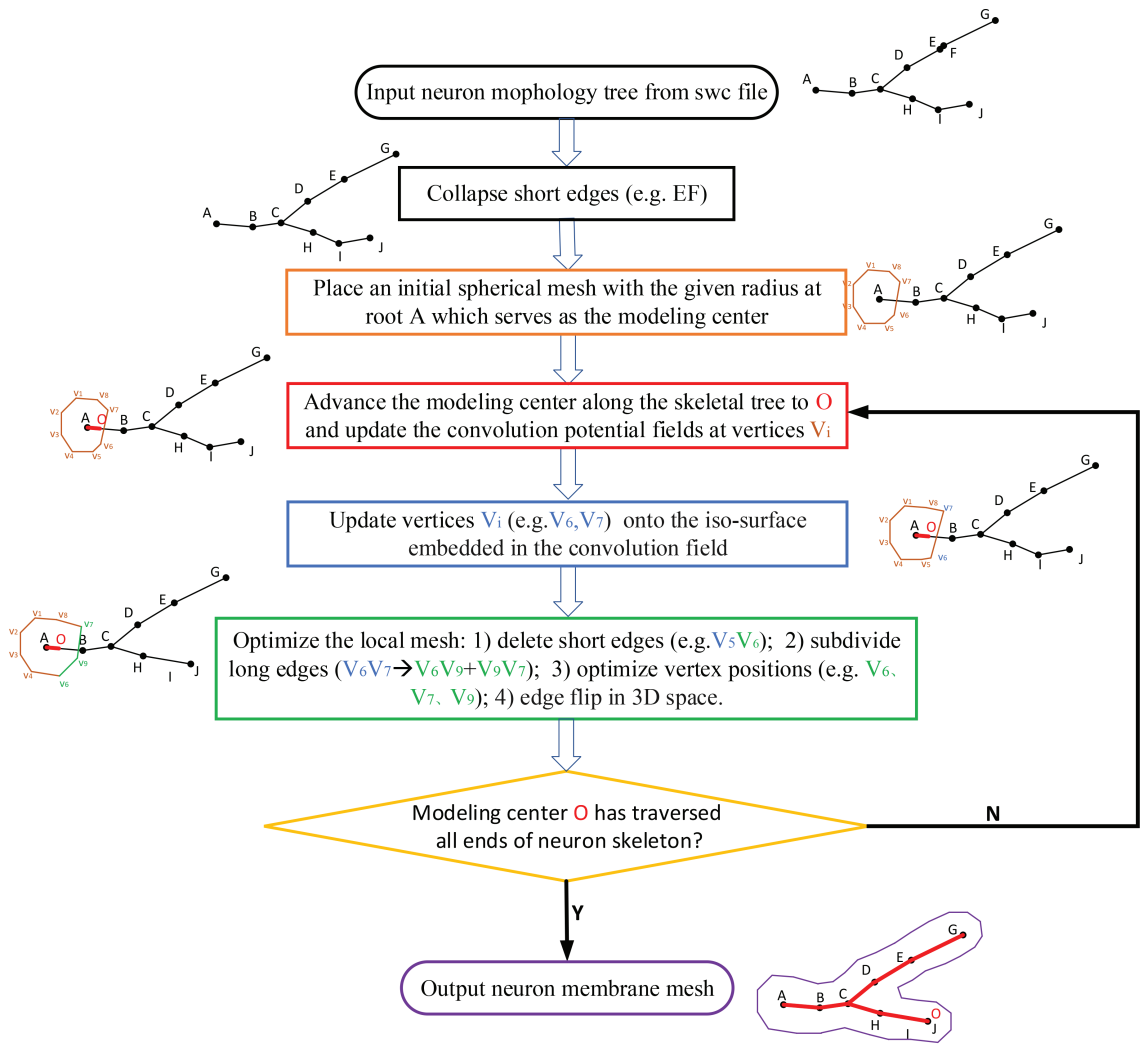
The workflow for the progressive meshing of neuronal morphology. The procedure starts from an initial soma mesh at the root of the neuron tree, then searches the vertices affected by the skeleton-based implicit surfaces, and finally projects these vertices onto the implicitly defined convolution surfaces for further remeshing if the relevant edges are longer than the predefined threshold.

The neuronal membrane mesh is gradually generated through iterative ROI (Region of Influence) queries of an initial model, weighted deformations and local topology reconstructions, which can be represented with vertices and triangles. The whole workflow consists of the following steps (see the flow chart in Figure 4).

1. Precondition the neuronal tree.
2. Traverse the neuronal tree from the soma location.
3. Query the ROI in skeletal mappings.
4. Stretch and deform the mesh in the affected area to the approximated positions.
5. Optimize the mesh vertex distribution using remeshing with adaptive resolutions according to various geometric details.
6. Iteratively perform the same steps for all neurite paths.

### 2.2. Morphology preconditioning

Our approach takes skeleton-based representation of neuronal trees (e.g., the SWC files) as input. A neuronal tree typically origins from the soma node, and consists of a number of neurite segments that further bifurcate at branching points. While such segments should have a reasonable length in order to faithfully and effectively portray the geometry of the neuron, it is not unusual to find neurons with overly short segments in common data repositories such as NeuroMorpho.Org. Such artifacts in a neuronal tree could not only lead to redundant representation and thus slow down the overall mesh generation speed, but cause malfunction of the approach as well if the length of a segment is even smaller than the marching step. Therefore, as a pre-processing step, we eliminate all such trivial segments whose length *l*_*seg*_ < ϵ · *R*_*neuro*_ in the neuronal tree (see Figure 4).

### 2.3. ROI query strategy based on skeletal mapping

**Figure 5:**
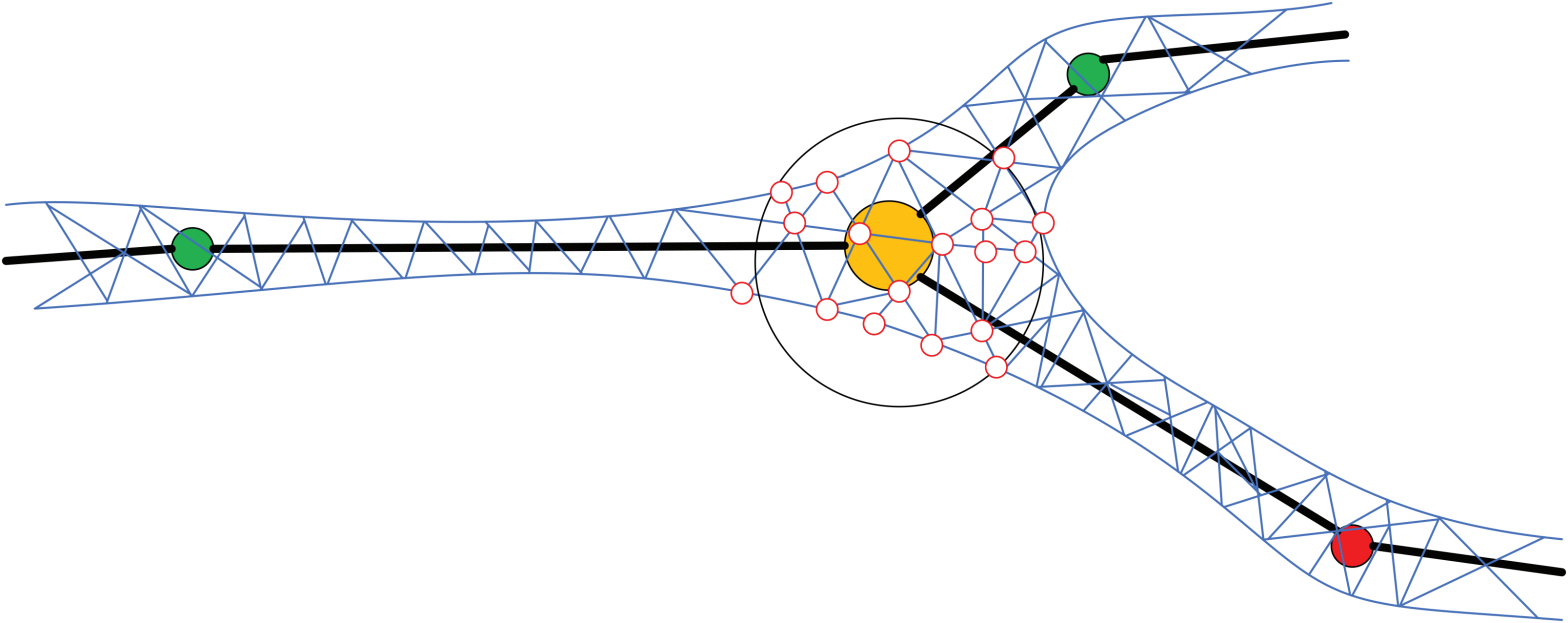
Mapping mesh vertices to the skeleton. For each position on the skeleton, an ROI (e.g., the hollow black sphere) is defined based on the local radius of the skeleton. All the mesh vertices inside of the ROI (e.g., the hollow red circles) are mapped to the current skeletal position and will be deformed accordingly.

In order to generate the neuronal membrane mesh, a skeletal mapping-based ROI strategy is proposed, which allows local deformation region queries. A list container is attached to each skeleton node for storing the indices of the vertices that are close to the current node. The mappings are created procedually, and the mesh vertex mappings are created based on the exact projection position of the current neuronal segment that deforms the vertex in question. We first query skeleton nodes in the ROI, and then query their mapping vertices on the membrane surface mesh. Compared with the ROI query algorithm based on space partition proposed in Zhu et al. (2013), our method further accelerates the generation of skeleton-driven mesh modeling. Figure 5 shows the ROI query of skeletal skins.

### 2.4. Local convolution surface approximation

Skeleton-based implicit surfaces are usually introduced to create branching structures (Bloomenthal and Shoemake, 1991; Hart and Baker, 1996; Jin and Tai, 2002) due to their smoothness and topological variations. However, the marching cubes polygonizations (Lorensen and Cline, 1987) of the iso-surface they employed suffer from high computation complexity, limited resolution, and low-quality triangular meshes. Furthermore, it is prone to missing small twigs for complex branch models because the output of the marching cubes is resolution-dependent. Even though there are a large number of improvements (Wyvill et al., 1986; Bloomenthal, 1994; Bottino et al., 1996; Akkouche and Galin, 2001; Van Overveld and Wyvill, 2004; Zhu et al., 2013), it is still difficult to balance the quality of the iso-surface polygons and the performance. Recently, a point skeleton-based metaball policy (Abdellah et al., 2020) is introduced to create accurate mesh models of brain vasculatures, and metaballs are also used to skin the different structural components of astrocytes (Abdellah et al., 2021) and then blend them in a seamless fashion. However, at straight line skeletons, too many metaballs have to be placed closely to approximate the cylindrical neuronal or vascular morphologies.

#### 2.4.1. Skeleton-based convolution surfaces

In this paper, our approach begins with an initial triangular mesh (soma), and progressively deforms it to approximate the target shape, which is defined as a convolution surface based on embedded neuronal line skeletons. Therefore, the final neuronal membrane surface mesh achieves high-order smoothness at arbitrary branches.

Convolution surfaces can be regarded as an isosurface embedded in a three-dimensional scalar field, which is calculated by convolving the geometric skeleton *V*_*i*_ with a low-pass filter function *f*. Given a skeleton segment:


(1) $$g_{i}(p)=\{\begin{array}{cc} 1, & p\in \text{ skeleton }V_{i}\\ 0, & \text{ otherwise } \end{array}$$

and a kernel function: *f*:*R*^3^ → *R*, the contribution of the potential value of the current skeleton in question at point *p* is the convolution of the function *f* and *g*_*i*_:


(2) $$F_{i}(p)=\int_{V_{i}}g_{i}(q)f(p-q)dV=(f⊗g_{i})(p)$$

#### 2.4.2. Finite support quartic kernel

Among them, the kernel function can be divided into infinite support (such as: Cauchy kernel function; McCormack and Sherstyuk, 1998) and finite support (such as: quartic polynomial kernel function; Jin et al., 2009). For a finitely supported kernel function, if the distance of a certain point from the skeleton is greater than the support radius of the kernel function, the potential contribution of the skeleton drops to zero. Instead, for an infinite support kernel function, no matter how far the point in the three-dimensional space is from the skeleton, the potential contribution of the skeleton to the current point is always greater than zero even though it is very tiny. As neuronal morphology usually appears to be a tree-like graph shape, we take line segments as the convolution skeleton, and the fourth-degree polynomial kernel function as the kernel function whose formula can be defined as:


(3) $$f_{\text{Quartic }}(r)=\{\begin{array}{cc} {\left(1-\frac{r^{2}}{R^{2}}\right)}^{2}, & r\leq R\\ 0, & r>R \end{array}$$

where *R* is the effective radius of the kernel function.

#### 2.4.3. Local convolution approximation

The convolution surface *S* based on a series of skeleton segments can be defined as:


(4) $$\begin{array}{l} S=\left\{p|\overset{n}{\underset{i=1}{\sum }}{\text{λ}}_{i}F_{i}\left(p\right)-T=0\right\} \end{array}$$

where *F*_*i*_ is the field contribution of the *i*^*th*^ skeletal segment as defined in Equation (2), λ_*i*_ is its weight factor, and *T* is the threshold for extracting the iso-surface from the embedded potential scalar field.

For a point *p* on the membrane mesh, the adopted local approximation scheme assumes that the closest skeleton to *p* is a line segment with infinite length. This assumption could not only reduce unnecessary convolution calculation beyond support radius, but also create natural blending between adjacent line segments due to their smooth thickness variations. Therefore, it is highly suitable for large amount of neuronal morphology approximation with complex graph structures. Actually, the potential contribution of an infinite skeleton to an arbitrary 3D position *p* can be calculated as:


(5) $$F_{\text{Quartic }}(p)=2{\text{λ}}_{i}\int_{0}^{\sqrt{R_{i}^{2}-d_{i}^{2}}}\left(1-\frac{d_{i}^{2}+x^{2}}{R_{i}^{2}}\right)\;dx=T$$

(6) $$\begin{array}{l} \Rightarrow {\text{λ}}_{i}=\frac{15TR_{i}^{4}}{16{\left(R_{i}^{2}-d_{i}^{2}\right)}^{\frac{5}{2}}} \end{array}$$

where *d*_*i*_ is the average distance between the *i*th skeleton and *p*, and *T* is still the global iso-value for the entire convolution surfaces; *R*_*i*_ is the effective radius of the kernel function corresponding to the current skeleton, which can be set to be an empirical value of 2*d*_*i*_ in our experiments; λ_*i*_ is the weight of the current skeleton segment, which can be derived analytically.

The solution to the above kernel function is based on the assumption that the value of the kernel function outside a certain distance is infinitesimal. Therefore, the fourth-order polynomial kernel function is usually preferred for its local support characteristics, which could reduce huge convolution computation.

#### 2.4.4. Convolution field-guided mesh projection

**Figure 6:**
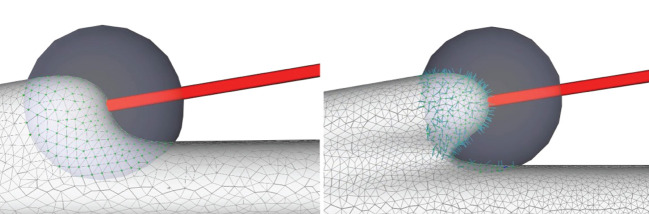
Convolution surface approximation at the branching regions using progressive evolution. In a branching region, a second neurite starts to grow from the main trunk **(left)**, and the implicit approximation and projection produce smooth structures **(right)**, which is the result of both deformation of the current neurite and blending of the neighboring branches.

The implementation of approximation is to project each vertex of the remeshed surface onto the implicit convolution surface in their respective gradient directions by performing standard Newton iterations (Vaillant et al., 2013):


(7) $$\begin{array}{l} p=p+sign\left(F\left(p\right)-T\right)\cdot l_{step}\cdot \frac{\text{Δ}F\left(p\right)}{|\text{Δ}F\left(p\right)|} \end{array}$$

where, the initial evolution step length *l*_*step*_ of the vertex *p* can be set as $step=\frac{1}{2}l_{e}$ and *l*_*e*_ is the length of the shortest edge coincident to *p*. After each subdivision, the projection step length of a new vertex can be derived from its adjacent vertices (Figure 6).

### 2.5. Remeshing algorithm based on quasi-uniform mesh structure

Isotropic remeshing is a valid scheme to optimize the distribution of mesh vertices and topological connectivity. Botsch et al. (2010) and Alliez et al. (2008) have reviewed various methods for surface remeshing. Numerous techniques like the particle-based methods (Ahmed et al., 2016), the Delaunay refinement methods (Cheng et al., 2013), and the randomized sampling methods (Ebeida et al., 2016) pay attention to generating isotropic meshes. The methods based on Centroidal Voronoi (Du et al., 1999) tessellation attempt to make each point in accord with the centroid of its Voronoi region. There are various ways to calculate a Voronoi diagram on surfaces, such as mesh parameterizations, discrete clustering, restricted Voronoi diagram (Yan and Wonka, 2015), and geodesic Voronoi diagram (Liu et al., 2016). The fundamental theory of the field-based methods is that isotropic triangular meshes can be taken out from sixway rotational symmetry directional fields (Du et al., 2018). In addition, there are edge-based approaches derived from local operators, including edge split, edge collapse, edge flip, and vertex repositioning (Botsch and Kobbelt, 2004; Dunyach et al., 2013; Wang et al., 2018).

Therefore, in our implicit mesh approximation, two significant aspects will be taken into consideration: (1) a triangular mesh representation of implicit surfaces should approximate the underlying geometry as accurate as possible, and (2) the mesh vertex distribution and connectivity should satisfy high-quality triangles with similar edge lengths coincident to the same polygon, allowing smooth membrane surface visualization and more stable voxelization for simulation.

**Figure 7:**
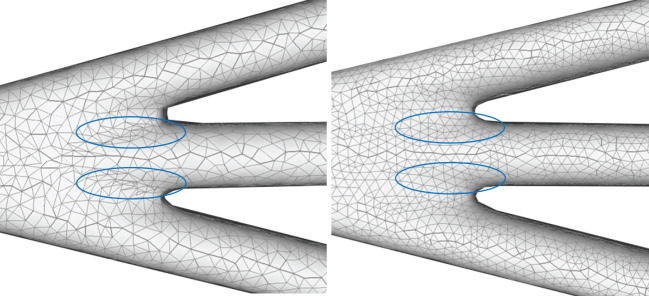
Quasi-uniform remeshing. The mesh without vertex optimization **(left)** includes many low-quality triangles especially near branching regions, as highlighted by the blue color. Our approach adjusts the unsatisfactory triangles by iterative remeshing optimization and generates quasi-uniform meshes **(right)**.

A quasi-uniform mesh (Stanculescu et al., 2011) supports successive surface deformation and subdivision at any level of detail, ensuring high-quality triangular mesh during the entire progressive deformation process. The details about how to build and remesh a quasi-uniform mesh will be given in the following subsections. Figure 7 illustrates the dynamic progressive mesh evolution with (right) and without (left) the adopted optimization operations. In the mesh generation, the remeshing based on quasi-uniform configuration can optimize the membrane mesh effectively, thereby achieving robust progressive modeling.

#### 2.5.1. Quasi-uniform mesh configuration

A uniform mesh is composed of polygon elements of roughly the same size, while a non-uniform mesh has elements of different sizes. In many aspects, uniform meshes enjoy such advantages as stable structures and efficient high-level geometric algorithms over non-uniform ones. However, creating uniform meshes is non-trivial especially for surfaces with complex topology. Therefore, a relaxation on constraints on uniform meshes is used here. As defined in Stanculescu et al. (2011), a mesh *M* is said to be quasi-uniform if there exist *l*_*T*_ and d, such that *M* results from iterative remeshing which assures a maximum edge length *l*_*T*_ and a minimum edge d. That is to say, for each edge *e* ∈ *edges*_*M*_, it should falls in the definite interval *length*_*e*_ ∈ (*d,l*_*T*_).

Given a closed manifold mesh *M* and a threshold *l*_*T*_, we can ensure that all edges of *M* are smaller than *l*_*T*_ by iteratively splitting the edges larger than *l*_*T*_, known as *l*_*T*_ tight mesh. The split operation amounts to adding a vertex at the midpoint of an edge, and correspondingly splitting the relevant triangle face into two adjacent triangles. The iteration over the edges is performed using a simple queue, and the resulting new edges are inserted into the queue.

The *l*_*T*_ tight property is not the only factor to ensure high quality triangles, since edge collapses should be performed to delete too short edges to generate higher-quality triangle meshes. A edge collapse operation moves the two vertices of a short edge to its midpoint to collapse the edge, ensuring that all the edges of *M* will not be shorter than the collapse threshold *d* (that is, the minimum edge length). Moreover, it is necessary to check the validity of the collapse operation before each implementation. If a collapse operation produces intersected triangles, the operation is illegal and will not be performed for the current edge.

Obviously, it is difficult to guarantee that the lengths of all the edges generated after the collapse operation are greater than the minimum length d, and the collapse operation may also destroy the *l*_*T*_ tightness property on the mesh. Therefore, before establishing the *l*_*T*_ tight mesh *M*, it is necessary to perform the collapse operation to ensure that the minimum length of the mesh edges is greater than d, and then the *l*_*T*_ tightness property should be restored. In fact, *d* can be taken as an internal parameter of a quasi-uniform mesh, since it is bound to *l*_*T*_:d <= *l*_*T*_/2, ensuring that the edges greater than *l*_*T*_ will not be divided into edges shorter than *d* while establishing the *l*_*T*_ tight mesh after a collapse operation.

#### 2.5.2. Local quasi-uniform mesh remeshing in ROI

Based on the quasi-uniform mesh configuration, too long or too short edges should be splitted or collapsed, respectively, which will be followed by mesh optimizations including vertex valence equalization, triangle area equalization, and implicit surface projection (see Algorithm 1).

**Algorithm 1:** Remeshing.

**Input:** *init*_*surface*, *target*_*edge*_*length*
**Output:** quasi-uniform mesh
1: **function** remesh(*init*_*surface, target*_*edge*_*length*)
2: *result*←*init*_*surface*
3: *low*←α*target_*edge*_*length*
4: *high*←β*target_*edge*_*length*
5: **for** *vHandle*:*vHandles* **do**
6: *spilt*_*long*_*edges*(*high*)
7: *collapse*_*short*_*edges*(*low, high*)
8: *equalize*_*valences*()
9: *tangential*_*relaxation*()
10: *project*_*to*_*surface*()
11: **end for**
12: **return** *result*
13: **end function**

##### 2.5.2.1. Vertex valence equalization

In order to optimize each triangle of a mesh as regularly as possible, an edge flip operation can be applied to achieve the vertex valence of 6. Similar to edge collapses, a legality check is also needed before each flip implementation.

##### 2.5.2.2. Triangle area equalization

After iteratively performing the above optimization operations on the mesh *M*, we can get a quasi-uniform triangular mesh. The edge lengths of the mesh are basically close to the target length *l*, which satisfies d < *l* < *l*_*T*_, and the valences of the vertices are close to 6. However, the triangle areas and the edge lengths may still vary with each other, which can be further optimized. Therefore, we carry out a vertex fine-tuning through an area-based tangential smoothing. Each vertex *p*_*j*_ is assigned a weight that equals to its averaged area *A*(*p*_*j*_), and a tangential smoothing moves *p*_*j*_ to its weighted barycentric position


(8) $$\begin{array}{l} g_{i}=\frac{1}{\sum_{p_{j}\in N\left(p_{i}\right)}A\;\left(p_{j}\right)}\underset{p_{j}\in N\left(p_{i}\right)}{\sum }A\;\left(p_{j}\right)p_{j}. \end{array}$$

##### 2.5.2.3. Iterative implicit surface projection and smoothing

**Figure 8:**
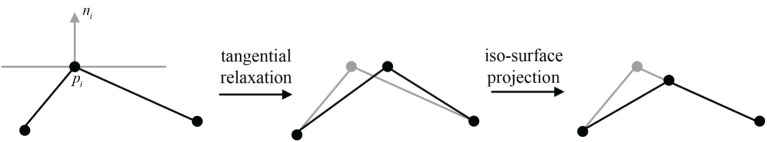
A 2D illustration for the update of *p*_*i*_'s position by tangential relaxation and iso-surface projection.

Following each tangential smoothing, a projection onto implicit surfaces can be performed for each smoothed vertex (Figure 8), otherwise jitter artifacts could occur. The new position can be updated according to the projection formula


(9) $$\begin{array}{l} p_{i}\leftarrow p_{i}+\text{λ}\left(I-n_{i}n_{i}^{T}\right)\left(g_{i}-p_{i}\right), \end{array}$$

where *n*_*i*_ is the normal vector and λ is the damping factor for oscillation avoidance. Vertices with relatively large Voronoi regions have more weight, attracting mesh vertices and thus reducing their own area. Experiments show that the mesh area of vertex-related region can be averaged without too many iterations.

### 2.6. Strategies for adaptive mesh vertex density

To ensure robust generation of high-quality mesh models for neuronal morphologies, a mesh optimization for high-resolution levels of details is required. However, a high-resolution representation usually results in too many tiny triangles on the surface parts with low curvatures, which not only imposes high burden on memory but also wastes unnecessary computational time. On the other hand, in order to generate topologically correct surfaces at branches robustly, a relative high-resolution surface mesh is preferred. Therefore, the marching step size of the mesh evolution should be related to both the skeletal structures and mesh vertex densities so as to create neuronal morphology with adaptive edge lengths.

**Figure 9:**
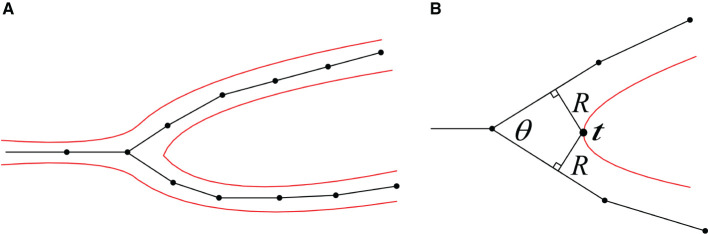
**(A)** A 2D illustration of a bifurcation and its corresponding iso-surface. **(B)** The calculation of the separating position *t*.

The robustness of the surface mesh generation at branches and the corresponding mesh vertex density depend on the angles between the branches, which are used to control the marching step size along the skeletons. Actually the mesh vertex density can be determined according to the target edge length in the current region, which is indirectly related to the branching angles. It should be noted that the mesh vertices in the ROI ought to be queried as fast as possible for efficient mesh evolution, so the step sizes of both the marching along skeletons and the convolution surface approximation should not exceed the lower length limit of the mesh edges. The details for determining vertex densities at a branch are as follows:

1. **Separating point**. According to the angle of a neuron branch and the support radius *R* of the target convolution surfaces, the separating position *t* at the branch can be pre-calculated. Since the distances between *t* and the two bifurcations are both *R*, the projection points of *t* on the skeleton segments can be deduced based on the branching angle θ, which correspond to the positions with the maximum vertex densities in dynamically adaptive mesh evolution (Figure 9).
2. **Maximum vertex density**. As the neuronal morphology at the branching *t* usually creates a surface with locally maximal curvature, the maximum vertex densities are assigned for the mesh in the nearby region. Therefore, triangles with the minimum edge length are generated in the separating region based on a marching along the skeleton with the minimum step size.
3. **Minimum vertex density**. On the other hand, the maximum target edge length can be usually set as the average edge length of the initial mesh, as our mesh evolution begins with the soma that has the largest radius. The local maximum edge lengths at other positions that are far away from branching can be calculated according to the ratio of the current skeletal radii and the soma radius.
4. **Edge length interpolation**. After the locally minimum target edge lengths and the maximum ones are determined, edge lengths for intermediate regions between each pair of the minimum *l*_min_ at *t* and maximum length *l*_max_ can be linearly interpolated accordingly.

**Figure 10:**
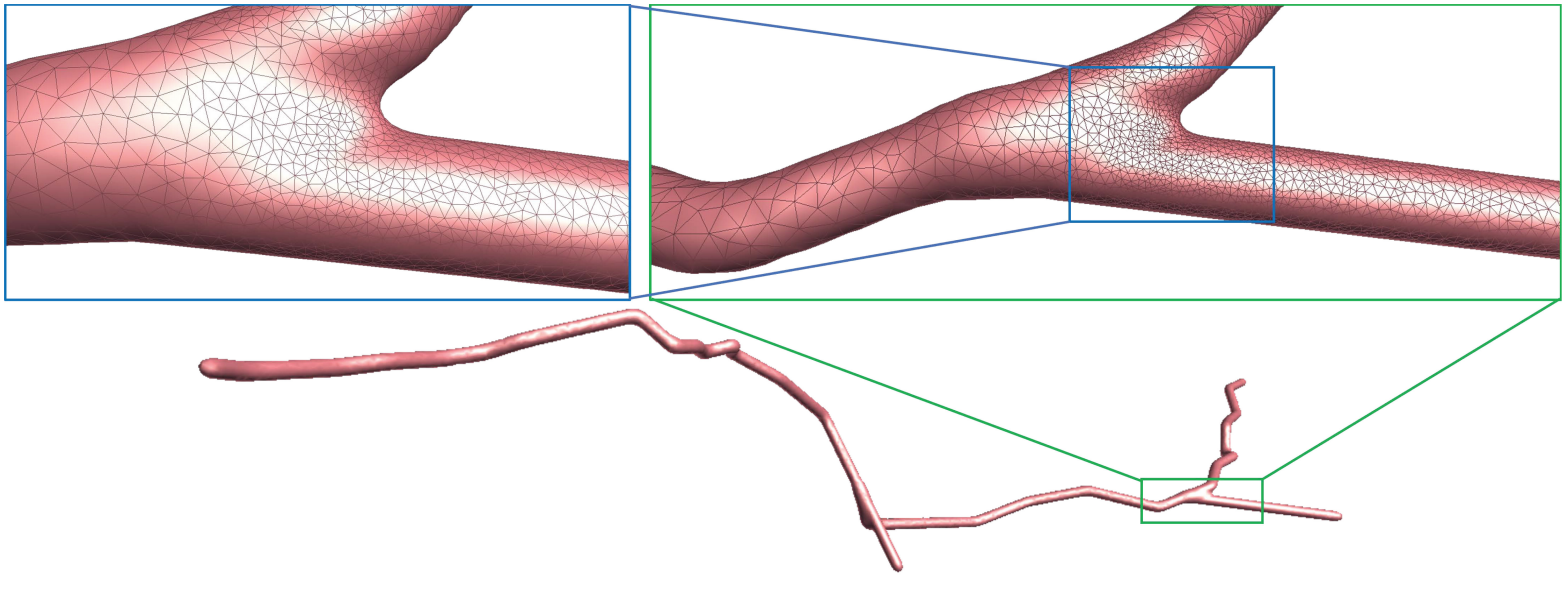
Adaptive mesh vertex distributions in a bifurcating region. High-density vertices are placed at the bifurcating region to effectively capture geometrical details.

As demonstrated in Figure 10, the adaptive mesh evolution makes the overall vertex distribution more reasonable. Moreover, such optimization also leads to accelerated surface generation and subsequent rendering.

## 3. Results and conclusion

### 3.1. Visual results

**Figure 11:**
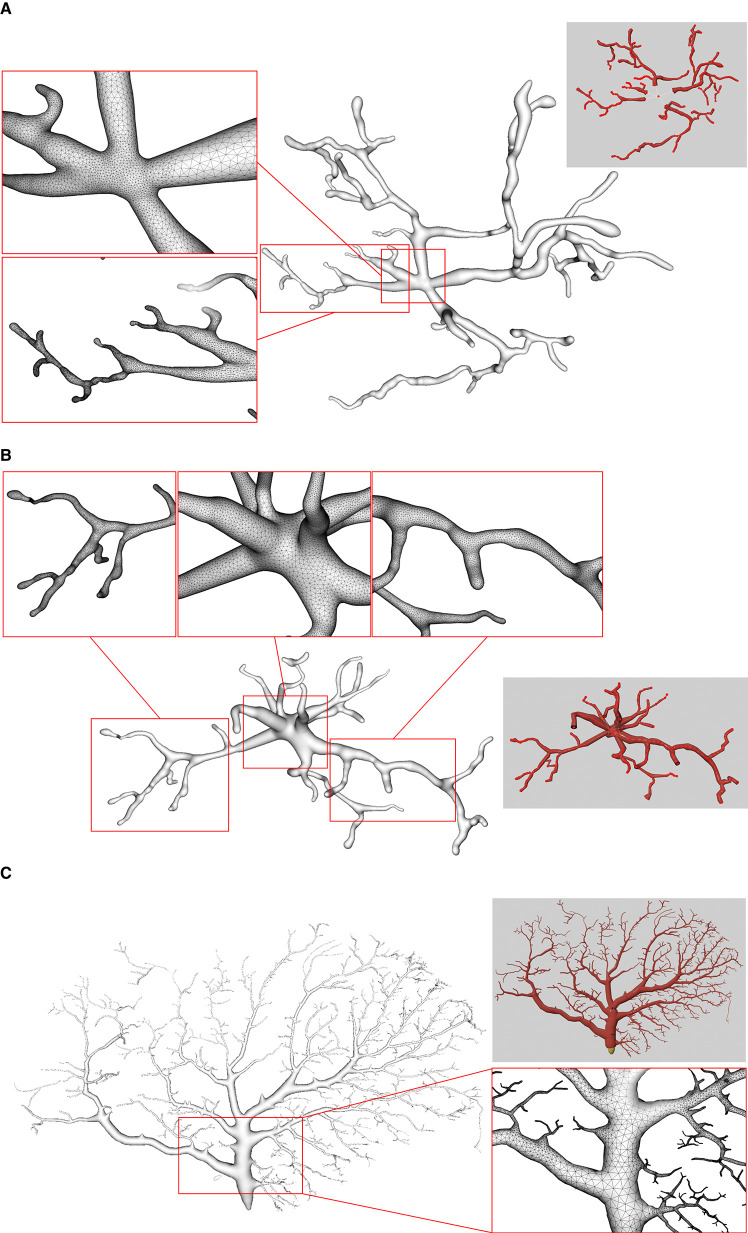
Examples of three surface meshes generated by the proposed approach. Local regions on the meshes are zoomed in for the display of details. The corresponding input point-and-diameter morphology data are shown in small panels. **(A)** A cell of a mouse, which includes 345 nodes and 61 branches. **(B)** A cell of a mouse, and it is composed of 247 nodes and 46 branches. **(C)** A cell of a drosophila melanogaster with 3224 nodes and 1169 branches.

We use several data from NeuroMorpho.Org, one of the largest cell morphology database, to validate our proposed approach, as shown in Figure 11. It can be seen that the underlying convolution potential field can well smooth the whole membrane surface. Due to the advantageous superposition property of convolution surfaces, crease-free rounded neuronal surfaces can be produced without any advanced blending operations. Moreover, in order to improve the efficiency of the modeling system, we propose the ROI query strategy based on skeleton-vertices mapping, which can effectively accelerate the mesh evolution.

**Figure 12:**
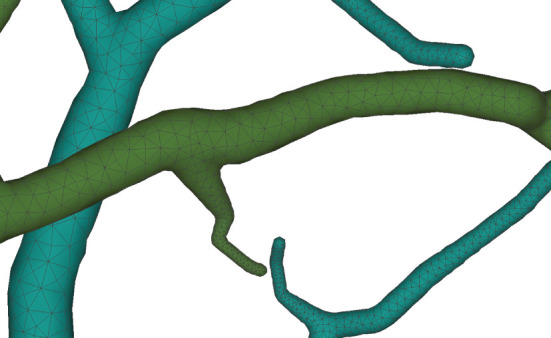
Membrane surfaces with adaptive edges for different thickness: surfaces with larger radius are created with vertices of lower densities, and vice versa.

**Figure 13:**
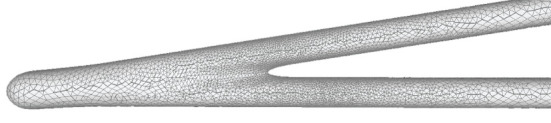
Adaptive vertex distribution at a bifurcating structure. Special care is taken at the separating position to create smooth high-curvature surfaces.

On the other hand, the mesh vertex density distribution is optimized to represent adaptive details. The density of the surface vertices is related to both the thickness (Figure 12) of the neuronal morphology and the surface curvature (Figure 13). Although the thickness can be easily obtained from the morphology files, it is non-trivial to calculate the curvature in a dynamic mesh evolution. The main reason lies in that the curvature has to be pre-determined before the quasi-uniform remeshing, which is used in the edge length limitations. To this end, our insight is that high curvatures are distributed at branching regions especially at the separating regions of skeleton-based convolution surfaces. Therefore, a proportionate ratio to the distance between an edge and the separating points is employed to the current edge length.

**Figure 14:**
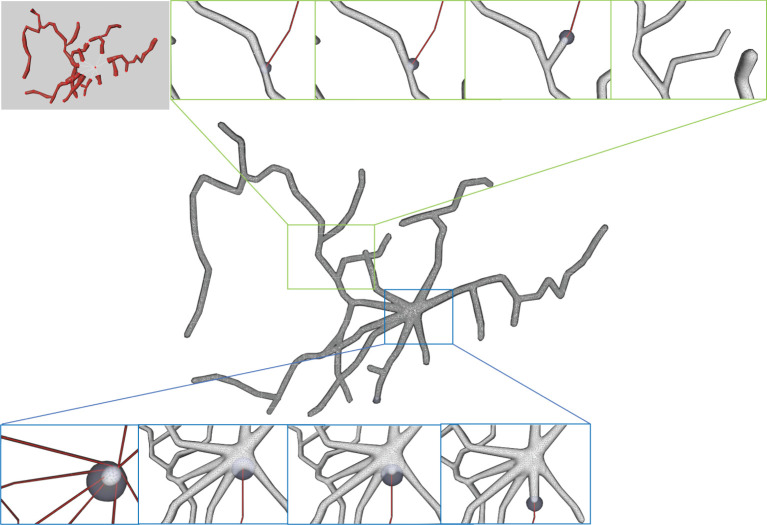
Progressive surface evolution. The mesh growing processes at a branch **(top)** and at the root node **(bottom)** are illustrated for several different timestamps.

As the final mesh is progressively deformed from an initial sphere (Figure 14), no deliberate stitching operations are involved. During the dynamic mesh evolution, a quasi-uniform mesh structure is used to guide the edge split and collapse, which prevents too long or too short edges that are detrimental to a robust mesh generation.

### 3.2. Comparisons

**Figure 15:**
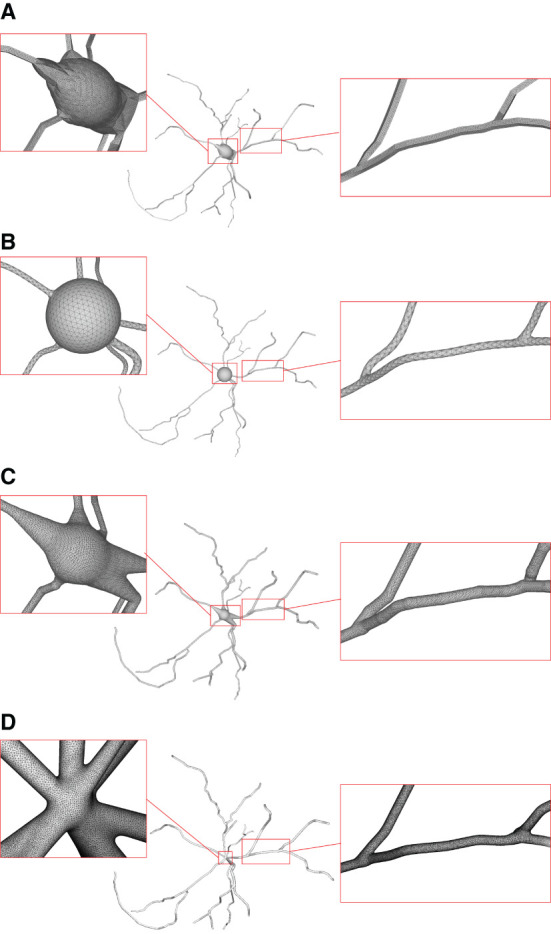
Comparison of the generation of a same neuron using different approaches. Models generated using NeuroTessMesh **(A)**, AnaMorph **(B)**, Neuromorphovis **(C)**, and our proposed method **(D)** are shown, respectively.

Neuronal membrane surface meshing is a fundamental preparation for neuronal electrical and chemical simulations. In Figure 15, we compare the mesh generation performance among our software and several other tools. Earlier software packages, such as Neurolucida (Glaser and Glaser, 1990), NeuroConstruct (Gleeson et al., 2007), NeuGen (Eberhard et al., 2006), and Genesis (Wilson et al., 1988) provide approximations of neuron surfaces with mesh-based methods. Unfortunately, they tend to create meshes with low qualities, in which self-intersected parts usually occur in branching regions. Other methods such as the one presented in Lasserre et al. (2011) start from a sphere (made with quads) with a fixed resolution, and the dendrites are then generated by quad-extrusions starting from the soma. At the end of the method, a Catmull-Clark subdivision is employed to smooth the whole mesh, generating realistic, smooth, and closed meshes. However, the preservation of desirable properties of being 2D-manifold is not stated as an objective in the work.

**CTNG** (McDougal et al., 2013) uses constructive solid geometry to define a plausible reconstruction without gaps, which represents a geometry as the unions and intersections of three-dimensional graphics primitives. It then uses “constructive cubes” to produce a watertight triangular mesh of the neuron surface. However, it depends on the underlying marching cubes, which create triangles with greatly varying aspect ratios.

**Neuroize** (Brito Menéndez et al., 2013) defines a physics-based mass-spring system to generate a neuron mesh, which could create a high-quality mesh. However, due to the versatility of the mass-spring system, complicated fine-tuning of several simulation parameters is required to achieve an accurate reconstruction.

**NeuroTessMesh** (Garcia-Cantero et al., 2017) improves the technique in Neuronize by applying a FEM (Finite Element Method) (Erleben et al., 2005) to simulate the deformation, which enables a convenient control over the mesh deformation. The approach approximates the cell bodies and the dendritic and axonal arbors in independent procedures that are later merged, resulting in a closed surface that approximates a whole neuron. However, NeuroTessMesh does not deal with mesh generation at branch points, and in particular, the junction between neurite and soma is not smooth, which is obviously not desirable as shown in Figure 15A. Although NeuroTessMesh can adapt mesh resolution based on the distance to the camera, it cannot adapt mesh resolution based on different geometric details when generating individual neuron mesh models.

**AnaMorph** (Mörschel et al., 2017) uses a recursive tessellation algorithm starting from an icosahedron to construct the soma mesh as an initial mesh. The AnaMorph framework can produce relatively high-quality meshes, but it is not robust for building some complex neuronal structures, especially for high resolution modeling at branches. Moreover, a complex stitching operation has to be performed on adjacent branches in order to produce a watertight surface (Figure 15B).

**Neuromorphovis** (Abdellah et al., 2018) extends an earlier meshing algorithm (Abdellah et al., 2017) and is capable of reconstructing piecewise watertight meshes that could be employed to visualize detailed electrophysiological activities obtained from voltage dynamics simulations. However, too many metaobjects have to be placed for creating a smooth-varying implicit field for extracting a neuronal membrane iso-surface. In addition, a post-optimization is required to improve the extracted triangles with greatly varying aspect ratios (Figure 15C).

**Our approach** takes the membrane surface mesh creation as a digital sculpting guided by the neuronal morphologies. It produces high-quality meshes (Figure 15D) robustly by deforming an initial mesh iteratively.

### 3.3. Quantitative evaluations

As our approach employs a progressive mesh generation, considerable time is required to complete the growing process. In spite of this, acceptable performance can be obtained for regular neuronal membrane mesh creations as shown by the timings listed in Table 1 for the examples used in our experiments. All the data are tested on a PC with a 2.9 GHz Intel Core i7-10700 CPU (only 1 core is used) with 16 GB memory. Although our approach is computation-intensive due to its procedural deformation, very few memories are enough for successful executions. Therefore, our system can be performed on regular PCs.

**Table 1:** Performance evaluation.

**Models**	**Figure 2**	**Figure 3**	**Figure 10**	**Figure 11A**	**Figure 11B**	**Figure 11C**	**Figure 14**
Skeleton nodes	217	10	35	345	247	3,324	108
Mesh vertices	293,556	3,880	4,652	99,383	69,749	346,819	174,445
Time (s)	2,608	21	166	2,720	2,092	6,647	821
Memory (Mb)	70.6	42.1	44.4	63.9	53.5	100	56.7

For 3D simulations, our produced membrane meshes can be directly passed into TetGen (Si, 2015) to generate 3D tetrahedrons without any post-processing in spite that post-processing such as laplacian smoothing and global anisotropic analysis can be beneficial to TetGen voxelization. In our experiments, seven of our created surface meshes could be successfully voxelized (Table 2).

**Table 2:** Voxelization using TetGen (Si, 2015).

	**Species**	**Global**	**Local**
Figure 2	Mouse	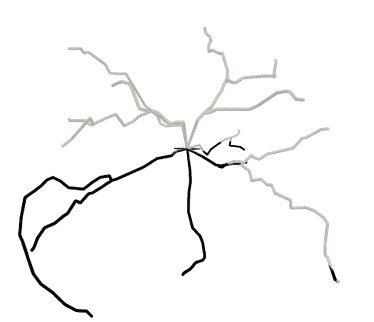	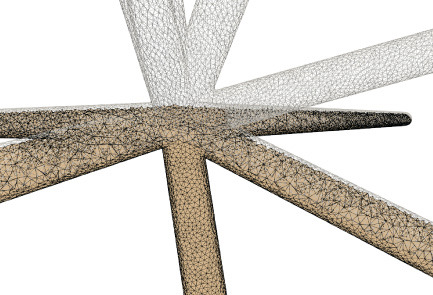
Figure 3	Mouse	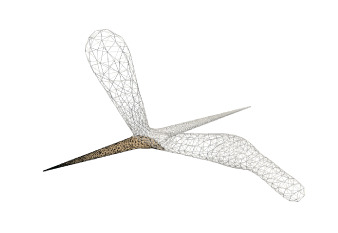	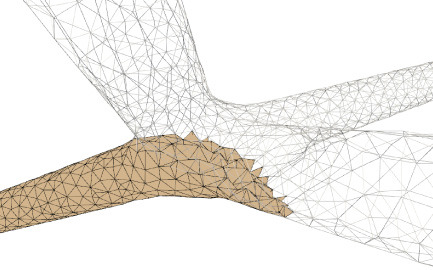
Figure 10	Zebrafish	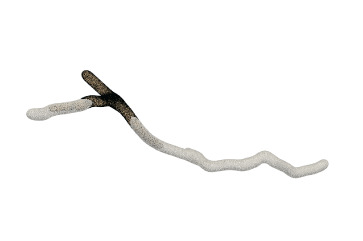	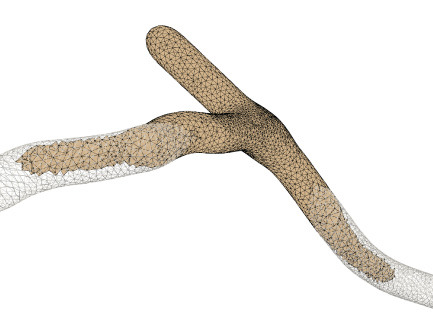
Figure 11A	Mouse	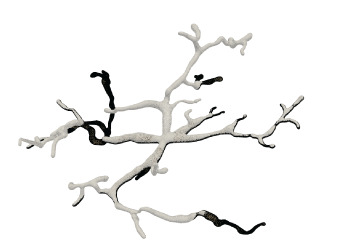	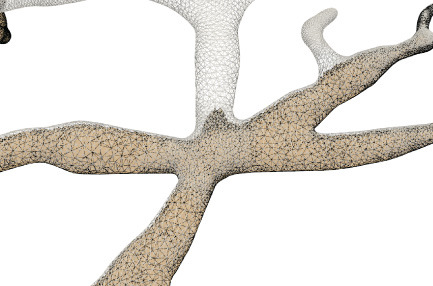
Figure 11B	Mouse	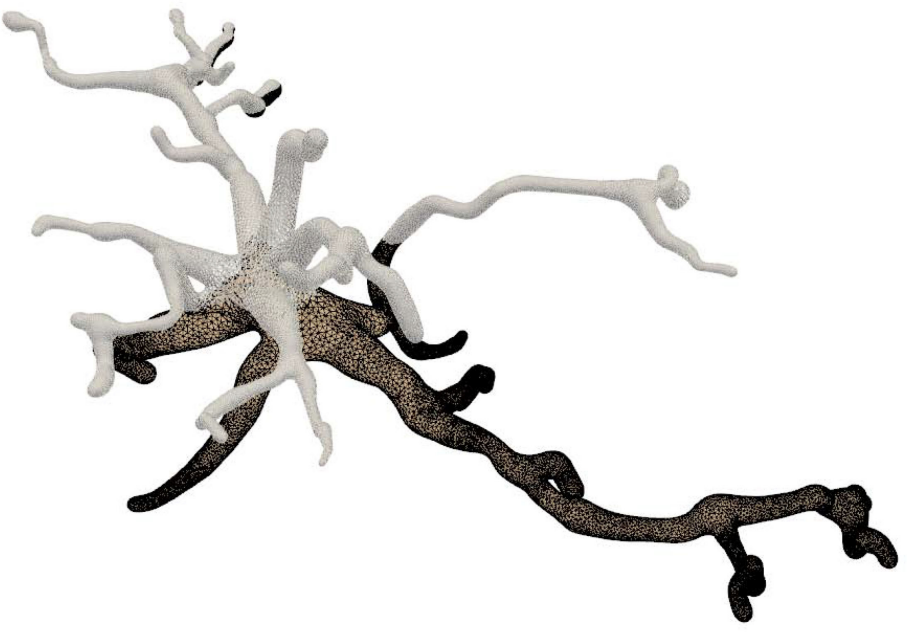	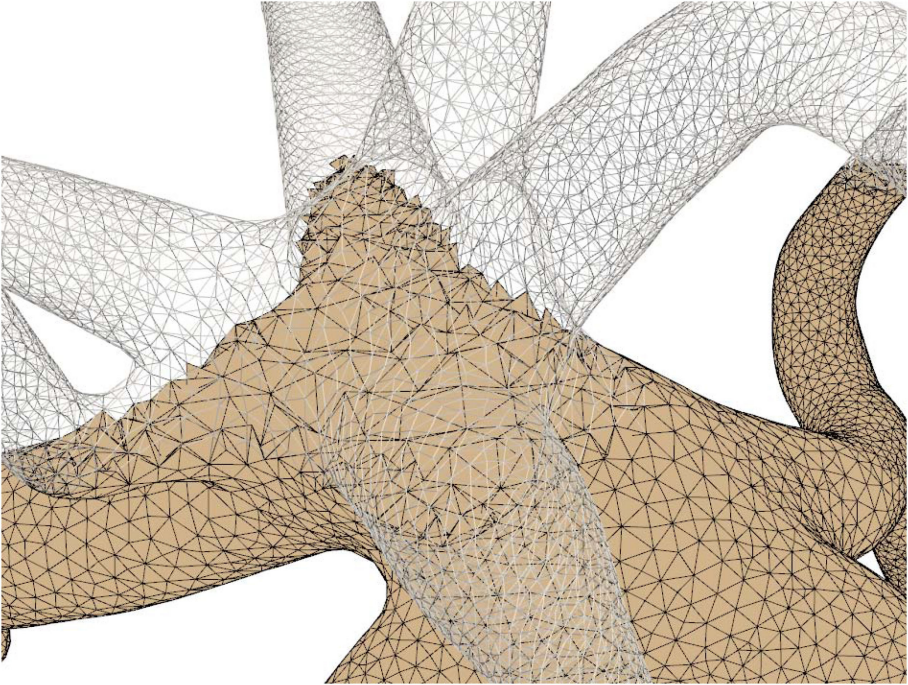
Figure 11C	Drosophila melanogaster	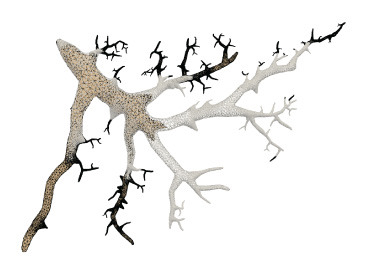	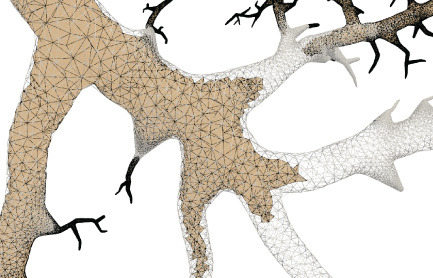
Figure 14	Mouse	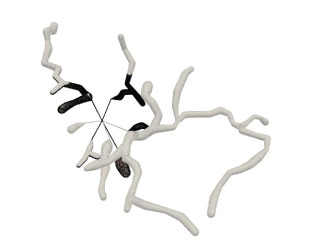	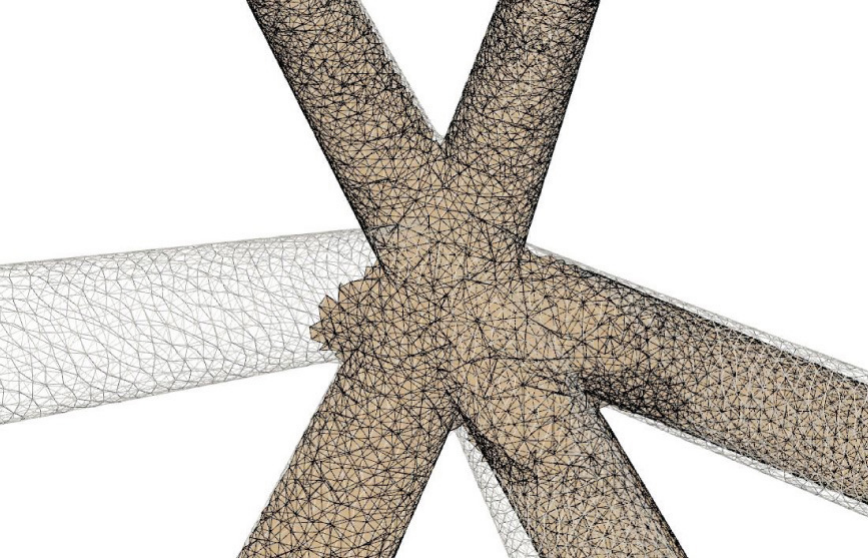

Finally, we processed more than 200 neuronal morphology data in batches from NeuroMorpho.Org for a more thorough evaluation of our approach, and achieved a success rate of 90%. In Table 3, attributes for both the morphology data and the generated meshes are given. It is worth mentioning that all of the produced meshes are manifold and watertight, with an average vertex valence of 6.

**Table 3:** Attributes of neuronal morphologies from NeuroMorpho.Org and produced membrane meshes averaged from groups of data.

**Morphology attributes**				**Membrane mesh attributes**					
**Archives**	**Species**	**Brain regions**	**# Models**	**# Avg. faces**	**Avg. face areas**	**# Avg. vertices**	**Avg. valence**	**Manifold (%)**	**Watertight (%)**
A. Mortensen	Rat	Neocortex	13	1,014,973	0.501	507,484	6	100.00	100.00
Aharon_Zuo	Mouse	Neocortex	12	1,063,122	0.934	531,562	6	100.00	100.00
Abrous	Mouse	Hippocampus	18	1,189,064	0.400	594,158	6	100.00	100.00
Acharya	Mouse	Hippocampus	15	1,303,338	0.728	674,744	6	100.00	100.00
Bock	Drosophila Melanogaster	Protocerebrum	15	1,875,781	0.270	959,308	6	100.00	100.00
Cardona	Drosophila Melanogaster	Protocerebrum	14	576,926	0.272	288,457	6	100.00	100.00
Baier	Zebrafish	Optic lobe	15	97,976	0.819	75,959	6	100.00	100.00
Borst	Drosophila Melanogaster	Optic lobe	20	877,115	3.226	438,454	6	100.00	100.00
Badea	Mouse	Retina	5	200,818	0.910	100,400	6	100.00	100.00
Baier	Zebrafish	Peripheral nervous system	5	71,776	1.094	35,890	6	100.00	100.00
Adori	Mouse	Peripheral nervous system	8	453,986	0.500	226,984	6	100.00	100.00
Badea	Mouse	Peripheral nervous system	11	453,986	1.488	226,984	6	100.00	100.00
Almuhtasib	Mouse	Basal ganglia	14	1,764,502	0.276	1,596,530	6	100.00	100.00
Anderson	Mouse	Basal ganglia	5	843,365	0.340	421,684	6	100.00	100.00
Alzheimer	Rat	Basal ganglia	11	1,100,110	1.664	550,028	6	100.00	100.00
Arenkiel	Mouse	Main olfactory bulb	5	502,111	0.284	251,068	6	100.00	100.00
Baier	Zebrafish	Main olfactory bulb	5	287,455	0.844	143,726	6	100.00	100.00
Adke_ Carrasquillo	Mouse	Amygdala	5	138,940	0.924	175,644	6	100.00	100.00
Argue	Rat	Amygdala	7	212,161	0.661	106,082	6	100.00	100.00

### 3.4. Conclusion and future work

This paper has presented a novel progressive approach for robustly generating high-quality 3D neuron models based on widely used point-and-diameter input of morphological tracings, such as those available in public repositories NeuroMorpho.Org. The final mesh is created by iteratively evolving an initial soma along the neuron skeletons. The adopted skeletal mapping policy assigns each vertex of the mesh to a definite skeletal node, which enables an efficient query of ROI. As described above, a sphere with uniformly distributed vertices is set as the initial soma for subsequent evolution, which performs dynamic local refinement, simplification and convolution surface approximation to generate a smooth neuronal membrane mesh with adaptive vertex density distribution. Therefore, the whole neuronal surface is always a closed 2D manifold mesh throughout all the evolution stages. Due to the superposition property of the adopted convolution surfaces, the soma surface can be automatically created based on the embedded skeletons, which are connected with each other at the soma. Actually the by-product of bumps at branches assists to create a plump shape without any special soma processing. In addition, to accelerate the computation-intensive convolution field generation, an analytical local convolution surface approximation is employed to approximate the line-skeletons in the form of point-and-diameter.

Neuronal membrane surfaces with high-quality meshes and smooth branches can be robustly achieved using our approach. Since the iterative deformation in our approach sacrifices the generation efficiency compared to a global mesh creation, advantageous superposition of convolution surfaces can be exploited to perform a “divide-and-conquer” policy for the whole neuron surface mesh generation in our future work. Moreover, a better soma can be modeled based on advanced physical simulation and contour-constrained implicit surface fitting. It is also worthwhile to extract a more precise soma model from raw volume data and use it as our initial evolution mesh. Finally, our created mesh model is a 2D manifold surface, which can be directly 3D printed for physical visualization and education purposes.

## Data availability statement

All the neuronal morphology data used in this work is made available at https://github.com/shugraphics/NeuroSkelMeshEvolution. Meshes generated from the morphology can be found from the same website. Besides, an executable program for Windows is also provided. Further inquiries can be directed to the corresponding author.

## Author contributions

XZ and YW contributed to conception and design of the study. XL programmed the functions of the project and wrote the first draft of the manuscript. SL and YS designed the workflow of the adopted algorithms and prepared the required testing data. LY optimized the adopted algorithm and analyzed the experimental results. All authors contributed to manuscript revision, read, and approved the submitted version.

## Funding

This study was supported by the National Natural Science Foundation of China (32071367), the Guangdong High Level Innovation Research Institute (2021B0909050004), the Natural Science Foundation of Shanghai (20ZR1420100), and the Key-Area Research and Development Program of Guangdong Province (2021B0909060002).

## Conflict of interest

The authors declare that the research was conducted in the absence of any commercial or financial relationships that could be construed as a potential conflict of interest.

## Publisher's note

All claims expressed in this article are solely those of the authors and do not necessarily represent those of their affiliated organizations, or those of the publisher, the editors and the reviewers. Any product that may be evaluated in this article, or claim that may be made by its manufacturer, is not guaranteed or endorsed by the publisher.

## Supplementary material

Video S1 Demo for progressive neuronal membrane surface generation.

## References

[B1] Abdellah M. Foni A. Zisis E. Guerrero N. R. Lapere S. Coggan J. S.. (2021). Metaball skinning of synthetic astroglial morphologies into realistic mesh models for *in silico* simulations and visual analytics. Bioinformatics 37(Suppl. 1), i426–i433. 10.1093/bioinformatics/btab280 34252950 PMC8275327

[B2] Abdellah M. Guerrero N. R. Lapere S. Coggan J. S. Keller D. Coste B.. (2020). Interactive visualization and analysis of morphological skeletons of brain vasculature networks with vessmorphovis. Bioinformatics 36(Suppl. 1), i534–i541. 10.1093/bioinformatics/btaa461 32657395 PMC7355309

[B3] Abdellah M. Hernando J. Antille N. Eilemann S. Markram H. Schürmann F. (2017). Reconstruction and visualization of large-scale volumetric models of neocortical circuits for physically-plausible *in silico* optical studies. BMC Bioinformatics 18:402. 10.1186/s12859-017-1788-4 28929974 PMC5606217

[B4] Abdellah M. Hernando J. Eilemann S. Lapere S. Antille N. Markram H.. (2018). Neuromorphovis: a collaborative framework for analysis and visualization of neuronal morphology skeletons reconstructed from microscopy stacks. Bioinformatics 34, i574–i582. 10.1093/bioinformatics/bty231 29949998 PMC6022592

[B5] Ahmed A. G. Guo J. Yan D.-M. Franceschia J.-Y. Zhang X. Deussen O. (2016). A simple push-pull algorithm for blue-noise sampling. IEEE Trans. Visual. Comput. Graph. 23, 2496–2508. 10.1109/TVCG.2016.2641963 28029623

[B6] Akkouche S. Galin E. (2001). Adaptive implicit surface polygonization using marching triangles. Comput. Graph. Forum 20, 67–80. 10.1111/1467-8659.00479

[B7] Alliez P. Ucelli G. Gotsman C. Attene M. (2008). “Recent advances in remeshing of surfaces,” in Shape Analysis and Structuring. Mathematics and Visualization, eds L. De Floriani and M. Spagnuolo (Berlin; Heidelberg: Springer), 53–82. 10.1007/978-3-540-33265-7_2

[B8] Bloomenthal J. (1994). An implicit surface polygonizer. Graph. Gems 4, 324–350. 10.1016/B978-0-12-336156-1.50040-929121077

[B9] Bloomenthal J. Shoemake K. (1991). “Convolution surfaces,” in Proceedings of the 18th Annual Conference on Computer Graphics and Interactive Techniques (Las Vegas, NV), 251–256. 10.1145/122718.122757

[B10] Botsch M. Kobbelt L. (2004). “A remeshing approach to multiresolution modeling,” in Proceedings of the 2004 Eurographics/ACM SIGGRAPH Symposium on Geometry Processing (New York, NY), 185–192. 10.1145/1057432.1057457

[B11] Botsch M. Kobbelt L. Pauly M. Alliez P. Lévy B. (2010). Polygon Mesh Processing. New York, NY: CRC Press. 10.1201/b10688

[B12] Bottino A. Nuij W. van Overveld C. (1996). “How to shrinkwrap a critical point: an algorithm for the adaptive triangulation of iso-surfaces with arbitrary topology,” in Proceedings of IS96, the Second Eurograhics/Siggraph Workshop on Implicit Surfaces (Eindhoven), 73.

[B13] Brito Menéndez J. P. Mata Fernández S. Bayona Beriso S. Pastor Pérez L. DeFelipe J. Benavides-Piccione R. (2013). Neuronize: a tool for building realistic neuronal cell morphologies. Front Neuroanat. 7:15. 10.3389/fnana.2013.00015 23761740 PMC3669758

[B14] Carnevale N. T. Hines M. L. (2006). The NEURON Book. Cambridge: Cambridge University Press. 10.1017/CBO9780511541612

[B15] Chen W. Liu M. Du H. Radojević M. Wang Y. Meijering E. (2022). Deep-learning-based automated neuron reconstruction from 3D microscopy images using synthetic training images. IEEE Trans. Med. Imaging 41, 1031–1042. 10.1109/TMI.2021.3130934 34847022

[B16] Cheng S.-W. Dey T. K. Shewchuk J. Sahni S. (2013). Delaunay Mesh Generation. Boca Raton, FL: CRC Press

[B17] Du Q. Faber V. Gunzburger M. (1999). Centroidal voronoi tessellations: applications and algorithms. SIAM Rev. 41, 637–676. 10.1137/S0036144599352836

[B18] Du X. Liu X. Yan D.-M. Jiang C. Ye J. Zhang H. (2018). Field-aligned isotropic surface remeshing. Comput. Graph. Forum 37, 343–357. 10.1111/cgf.13329

[B19] Dunyach M. Vanderhaeghe D. Barthe L. Botsch M. (2013). “Adaptive remeshing for real-time mesh deformation,” in Eurographics 2013 (Girona: The Eurographics Association).

[B20] Ebeida M. S. Rushdi A. A. Awad M. A. Mahmoud A. H. Yan D.-M. English S. A.. (2016). Disk density tuning of a maximal random packing. Comput. Graph. Forum 35, 259–269. 10.1111/cgf.12981 27563162 PMC4994978

[B21] Eberhard J. P. Wanner A. Wittum G. (2006). Neugen: a tool for the generation of realistic morphology of cortical neurons and neural networks in 3D. Neurocomputing 70, 327–342. 10.1016/j.neucom.2006.01.028

[B22] Erleben K. Sporring J. Henriksen K. Dohlmann H. (2005). Physics-Based Animation. Hingham, MA: Charles River Media Hingham.

[B23] Garcia-Cantero J. J. Brito J. P. Mata S. Bayona S. Pastor L. (2017). Neurotessmesh: a tool for the generation and visualization of neuron meshes and adaptive on-the-fly refinement. Front. Neuroinformatics 11:38. 10.3389/fninf.2017.00038 28690511 PMC5479896

[B24] Glaser J. R. Glaser E. M. (1990). Neuron imaging with neurolucida-a pc-based system for image combining microscopy. Comput. Med. Imaging Graph. 14, 307–317. 10.1016/0895-6111(90)90105-K 2224829

[B25] Gleeson P. Steuber V. Silver R. A. (2007). neuroConstruct: a tool for modeling networks of neurons in 3D space. Neuron 54, 219–235. 10.1016/j.neuron.2007.03.025 17442244 PMC1885959

[B26] Hart J. C. Baker B. (1996). “Implicit modeling of tree surfaces,” in Proceedings of Implicit Surfaces' 96 (Eindhoven), 143–152.

[B27] Jin X. Tai C.-L. (2002). Analytical methods for polynomial weighted convolution surfaces with various kernels. Comput. Graph. 26, 437–447. 10.1016/S0097-8493(02)00087-0

[B28] Jin X. Tai C.-L. Zhang H. (2009). Implicit modeling from polygon soup using convolution. Vis. Comput. 25, 279–288. 10.1007/s00371-008-0267-3

[B29] Kil Y. J. Renzulli P. Kreylos O. Hamann B. Monno G. Staadt O. G. (2006). 3D warp brush modeling. Comput. Graph. 30, 610–618. 10.1016/j.cag.2006.03.014

[B30] Lasserre S. Hernando J. Hill S. Schuermann F. de Miguel Anasagasti P. Abou Jaoudé G.. (2011). A neuron membrane mesh representation for visualization of electrophysiological simulations. IEEE Trans. Visual. Comput. Graph. 18, 214–227. 10.1109/TVCG.2011.55 21383404

[B31] Liu Y.-J. Xu C. Yi R. Fan D. He Y. (2016). Manifold differential evolution (MDE): a global optimization method for geodesic centroidal voronoi tessellations on meshes. ACM Trans. Graph. 35, 243–251. 10.1145/2980179.2982424

[B32] Lorensen W. E. Cline H. E. (1987). “Marching cubes: a high resolution 3D surface construction algorithm,” in Proceedings of the 14th Annual Conference on Computer Graphics and Interactive Techniques, SIGGRAPH 1987 (Anaheim, CA), 163–169. 10.1145/37401.37422

[B33] McCormack J. Sherstyuk A. (1998). Creating and rendering convolution surfaces. Comput. Graph. Forum 17, 113–120. 10.1111/1467-8659.00232

[B34] McDougal R. A. Hines M. L. Lytton W. W. (2013). Water-tight membranes from neuronal morphology files. J. Neurosci. Methods 220, 167–178. 10.1016/j.jneumeth.2013.09.011 24091136 PMC4197804

[B35] Mörschel K. Breit M. Queisser G. (2017). Generating neuron geometries for detailed three-dimensional simulations using anamorph. Neuroinformatics 15, 247–269. 10.1007/s12021-017-9329-x 28447297

[B36] Si H. (2015). Tetgen, a delaunay-based quality tetrahedral mesh generator. ACM Trans. Math. Softw. 41, 11:1–11:36. 10.1145/2629697

[B37] Stanculescu L. Chaine R. Cani M.-P. (2011). Freestyle: sculpting meshes with self-adaptive topology. Comput. Graph. 35, 614–622. 10.1016/j.cag.2011.03.033

[B38] Vaillant R. Barthe L. Guennebaud G. Cani M. Rohmer D. Wyvill B.. (2013). Implicit skinning: real-time skin deformation with contact modeling. ACM Trans. Graph. 32, 125:1–125:12. 10.1145/2461912.2461960

[B39] Van Overveld K. Wyvill B. (2004). Shrinkwrap: An efficient adaptive algorithm for triangulating an ISO-surface. Vis. Comput. 20, 362–379. 10.1007/s00371-002-0197-4

[B40] Vorsatz J. Ros¨sl C. Seidel H.-P. (2003). Dynamic remeshing and applications. J. Comput. Inf. Sci. Eng. 3, 338–344. 10.1115/1.1631021

[B41] Wang Y. Li Q. Liu L. Zhou Z. Ruan Z. Kong L.. (2019). TeraVR empowers precise reconstruction of complete 3-D neuronal morphology in the whole brain. Nat. Commun. 10, 3474. 10.1038/s41467-019-11443-y 31375678 PMC6677772

[B42] Wang Y. Yan D.-M. Liu X. Tang C. Guo J. Zhang X. Wonka P. (2018). Isotropic surface remeshing without large and small angles. IEEE Trans. Visual. Comput. Graph. 25, 2430–2442. 10.1109/TVCG.2018.2837115 29994531

[B43] Wilson M. Bhalla U. Uhley J. Bower J. (1988). “Genesis: a system for simulating neural networks,” in Advances in Neural Information Processing Systems (San Francisco, CA).

[B44] Wyvill G. McPheeters C. Wyvill B. (1986). “Soft objects,” in Advanced Computer Graphics, ed T. L. Kunii (Tokyo: Springer), 113–128. 10.1007/978-4-431-68036-9_8

[B45] Yan D.-M. Wonka P. (2015). Non-obtuse remeshing with centroidal voronoi tessellation. IEEE Trans. Visual. Comput. Graph. 22, 2136–2144. 10.1109/TVCG.2015.2505279 26661470

[B46] Zhu X. Guo X. Jin X. (2013). Efficient polygonization of tree trunks modeled by convolution surfaces. Sci. China Inform. Sci. 56, 1–12. 10.1007/s11432-013-4790-0

